# Bioinformatics and DNA-extraction strategies to reliably detect genetic variants from FFPE breast tissue samples

**DOI:** 10.1186/s12864-019-6056-8

**Published:** 2019-09-02

**Authors:** Aditya Vijay Bhagwate, Yuanhang Liu, Stacey J. Winham, Samantha J. McDonough, Melody L. Stallings-Mann, Ethan P. Heinzen, Jaime I. Davila, Robert A. Vierkant, Tanya L. Hoskin, Marlene Frost, Jodi M. Carter, Derek C. Radisky, Julie M. Cunningham, Amy C. Degnim, Chen Wang

**Affiliations:** 1 Departments of Health Science Research, Mayo Clinic, 200 1st Street SW, Rochester, MN 55905 USA; 2 Departments of Laboratory Medicine and Pathology, Mayo Clinic, 200 1st Street SW, Rochester, MN 55905 USA; 3 Departments of Neuroscience, Mayo Clinic, 4500 San Pablo Road, Jacksonville, FL 32224 USA; 4 Departments of Medical Oncology, Mayo Clinic, 200 1st Street SW, Rochester, MN 55905 USA; 5 Departments of Cancer Biology, Mayo Clinic, 4500 San Pablo Road, Jacksonville, FL 32224 USA; 6 Departments of Surgery, Mayo Clinic, 200 1st Street SW, Rochester, MN 55905 USA

**Keywords:** DNA sequencing, Target sequencing panel, Formalin-fixed tissue, Breast tissue, Mutational signature, Molecular barcode, Variant filtering

## Abstract

**Background:**

Archived formalin fixed paraffin embedded (FFPE) samples are valuable clinical resources to examine clinically relevant morphology features and also to study genetic changes. However, DNA quality and quantity of FFPE samples are often sub-optimal, and resulting NGS-based genetics variant detections are prone to false positives. Evaluations of wet-lab and bioinformatics approaches are needed to optimize variant detection from FFPE samples.

**Results:**

As a pilot study, we designed within-subject triplicate samples of DNA derived from paired FFPE and fresh frozen breast tissues to highlight FFPE-specific artifacts. For FFPE samples, we tested two FFPE DNA extraction methods to determine impact of wet-lab procedures on variant calling: QIAGEN QIAamp DNA Mini Kit (“QA”), and QIAGEN GeneRead DNA FFPE Kit (“QGR”). We also used negative-control (NA12891) and positive control samples (Horizon Discovery Reference Standard FFPE). All DNA sample libraries were prepared for NGS according to the QIAseq Human Breast Cancer Targeted DNA Panel protocol and sequenced on the HiSeq 4000. Variant calling and filtering were performed using QIAGEN Gene Globe Data Portal. Detailed variant concordance comparisons and mutational signature analysis were performed to investigate effects of FFPE samples compared to paired fresh frozen samples, along with different DNA extraction methods.

In this study, we found that five times or more variants were called with FFPE samples, compared to their paired fresh-frozen tissue samples even after applying molecular barcoding error-correction and default bioinformatics filtering recommended by the vendor. We also found that QGR as an optimized FFPE-DNA extraction approach leads to much fewer discordant variants between paired fresh frozen and FFPE samples. Approximately 92% of the uniquely called FFPE variants were of low allelic frequency range (< 5%), and collectively shared a “C > T|G > A” mutational signature known to be representative of FFPE artifacts resulting from cytosine deamination. Based on control samples and FFPE-frozen replicates, we derived an effective filtering strategy with associated empirical false-discovery estimates.

**Conclusions:**

Through this study, we demonstrated feasibility of calling and filtering genetic variants from FFPE tissue samples using a combined strategy with molecular barcodes, optimized DNA extraction, and bioinformatics methods incorporating genomics context such as mutational signature and variant allelic frequency.

**Electronic supplementary material:**

The online version of this article (10.1186/s12864-019-6056-8) contains supplementary material, which is available to authorized users.

## Background

High-throughput genomic and molecular characterizations of human tissues have propelled research and clinical care into the modern era of molecular medicine. This is particularly true in the field of breast cancer care, with mainstream use of molecular assays such as Mammaprint and OncotypeDX for clinical research and applications [1, 2]. Discovery efforts for molecular panels are best accomplished with nucleic acids derived from fresh or fresh frozen tissues, where the quality of nucleic acids is optimal. However, fresh or fresh frozen tissues are not always available for clinical research cohorts. In situations where molecular predictors are desired for long-term outcomes, the only available tissues are often archived formalin fixed paraffin embedded (FFPE) tissues from the past. Successful molecular and genomics measurements from FFPE tissues have remained elusive, due to impaired quality of DNA and RNA extracted from FFPE tissue blocks. Formalin fixation causes problems with nucleic acid structure, including fragmentation and cytosine deamination, and nucleic acids extracted from FFPE tissues are known to be of poor quality, resulting in questions regarding validity of results with next generation sequencing (NGS) from FFPE tissues [3–6]. For example, our research goal is to identify molecular and genetic markers of future breast cancer from benign breast biopsy tissues that were archived years prior to the cancer event [7]. In order to facilitate this effort, we are pursuing methodologic approaches to optimize DNA sequencing from FFPE tissues. In this report we describe an approach to evaluate paired FFPE and frozen DNA simultaneously to confirm validity of the sequencing results from FFPE tissues.

Other than FFPE DNA quality and quantity issues, the influence of FFPE sample preparation and extraction methods on downstream NGS data may also impact performance in downstream procedures. This is especially true when working with FFPE samples where age, fixation and storage may lead to artefactual results. To determine the effects of sample preparation on variant calling and downstream analysis we designed a pilot study, which included within subject replicates from breast tissue extracted using two different FFPE extraction methods. One of the methods included an enzymatic repair step that helps to reduce false mutation frequencies by removing artefactual C-T conversions that result from deamination during formalin fixation. Due to FFPE’s degraded nature, low concentration and need for high specificity, we chose a targeted amplicon-based sequencing approach, which was specifically designed to enrich gene targets relevant to breast cancer, and includes a unique molecular barcoding system that helps to remove duplicates and detect low frequency variants [8].

In this study, we thoroughly investigated variant calling concordance between paired FFPE and fresh-frozen samples, examined the allelic frequency distribution of called variants, and summarized mutational signatures specific to different DNA extraction methods for FFPE samples. The concordance of variant calls was also used to evaluate the expected false discovery rate (FDR) in FFPE samples and design practical strategies to prioritize variants from FFPE NGS products.

## Results

**Fig. 1:**
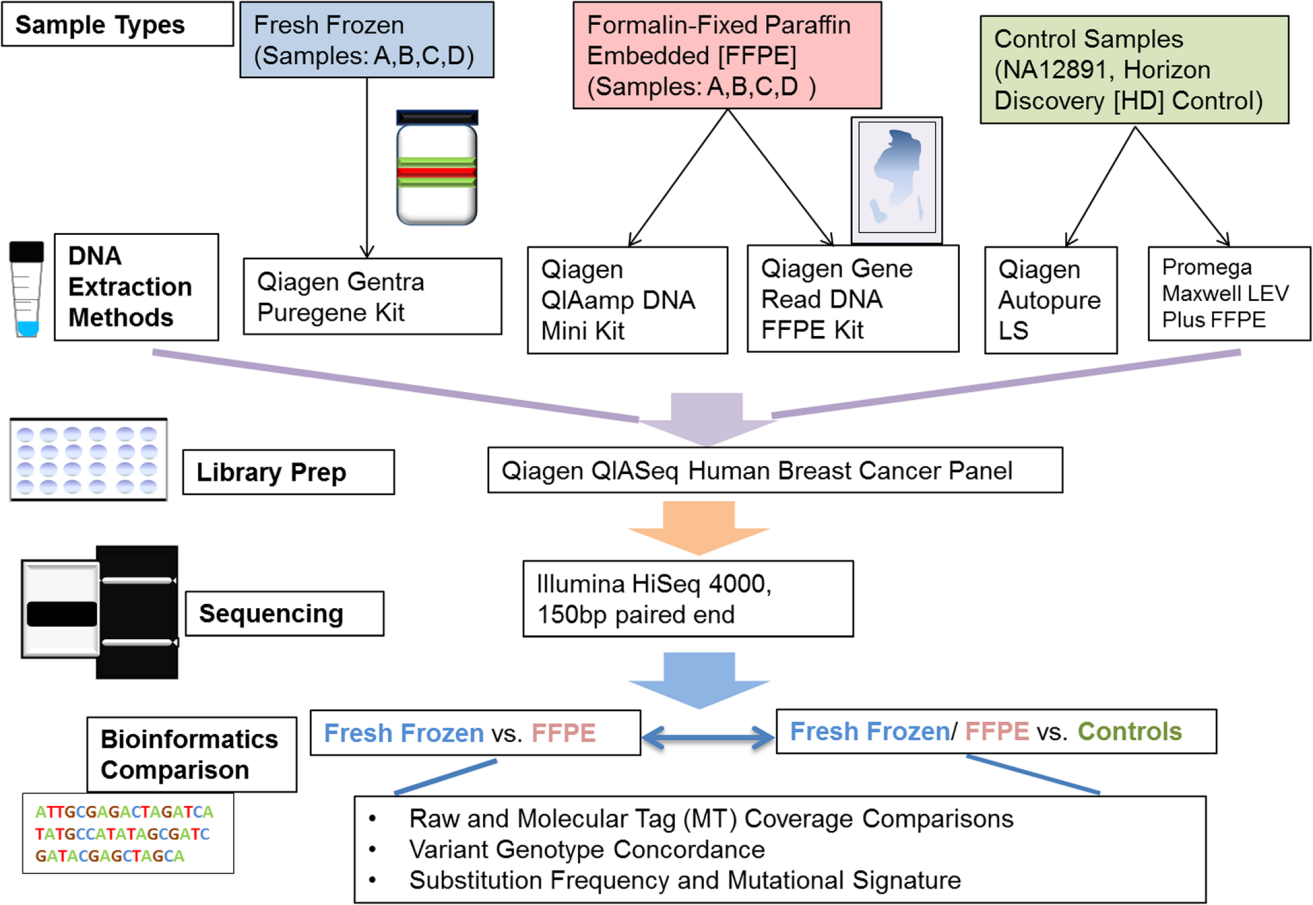
Overall workflow of the study: Along with positive and negative control samples, paired FFPE and fresh frozen samples went through different DNA extraction methods but same library preparation steps for Qiagen Breast Cancer Panel. After sequencing, bioinformatics analysis was conducted to examine coverages, variant calling and mutational signatures of called variants

**Fig. 2:**
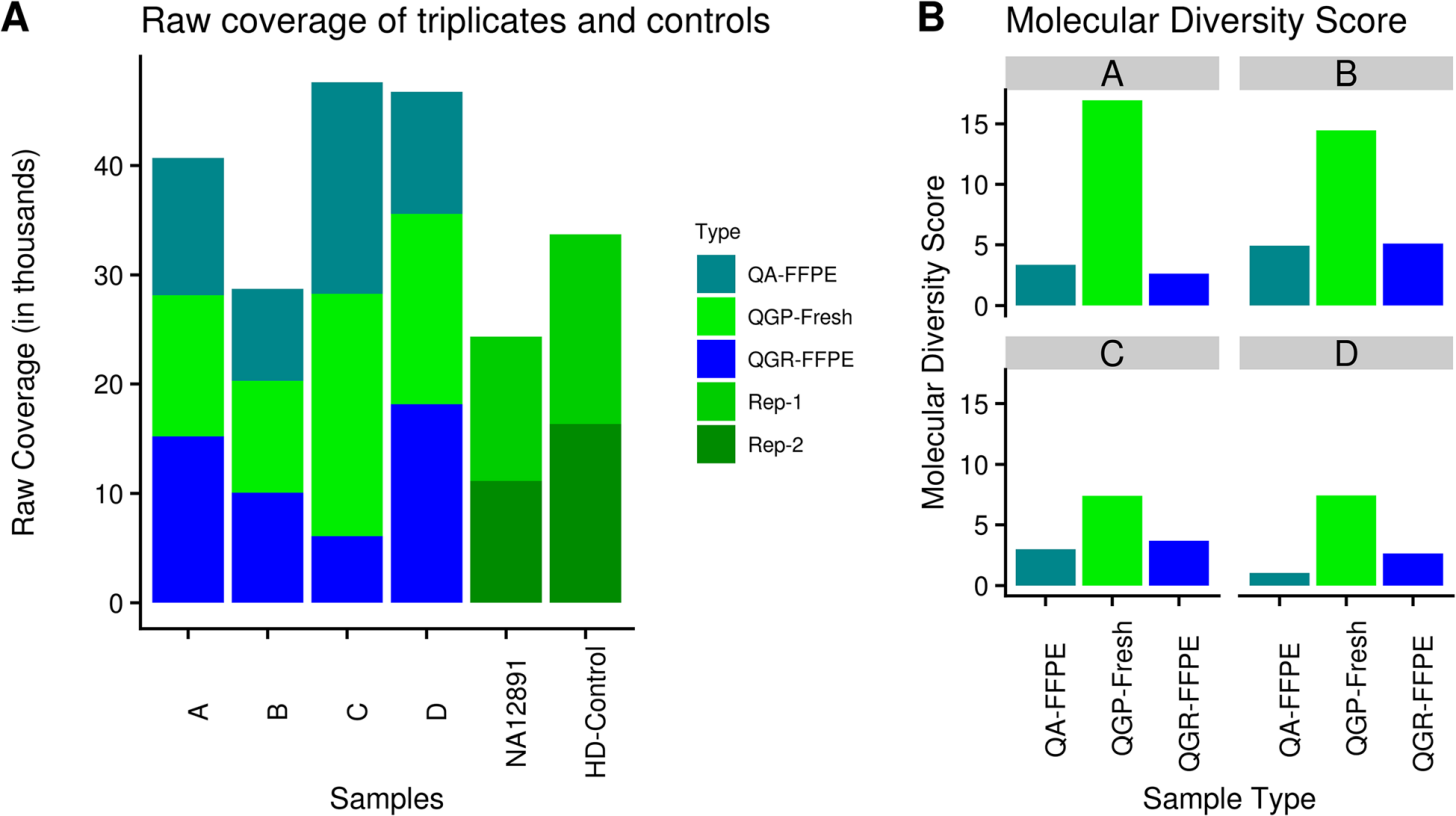
The sequencing coverages for triplicates, positive- (HD) and negative- (NA12891) control samples. **a.** Barplot of raw sequencing coverage; **b.** Barplot of the ratio of raw coverage to Molecular Tag (MT) Coverage; in which, the MT coverage is determined by collapsing all reads having the same unique molecular tag to a single consensus read

Figure 1 shows the overall workflow of this study, in which we systematically compared variant-calling results for four paired FFPE-frozen breast tissues, along with the well characterized negative and positive control samples. The variant results are also coupled with evaluations of two different FFPE DNA preparation kits: QIAGEN QIAamp DNA Mini Kit (“QA”) and QIAGEN GeneRead DNA FFPE kit (“QGR”), in order to evaluate the impacts of DNA extraction protocols in sequencing products and resulting variant calling results. All the samples were sequenced according to the QIAGEN breast cancer panel protocol, with molecular tag design to improve variant calling accuracy, and analyzed using the QIAGEN bioinformatics pipeline [8] and additional bioinformatics processes as described in the ‘Analysis’ section. Sample identify was successfully validated based on genotype concordance using NGScheckmate (Additional file 1). According to overall coverage evaluations, FFPE and frozen samples had overall comparable raw coverage (Fig. 2a), but FFPE samples’ MT coverage were consistently lower than their frozen pairs due to lower molecular diversity scores for FFPE samples (Additional file 2). Noticeable MT-coverage differences can also be seen between the negative-control genomic sample (NA12891) and the positive control sample with intended FFPE degradation effect (HD-Control). As MT-coverage is quantified by collapsing sequencing reads sharing the same original molecular barcode before PCR amplification, this indicates FFPE samples had significantly lower DNA molecule diversity than their frozen pairs, supported by much lower proportions of MT-coverage versus raw NGS coverage, i.e. lower molecular diversity scores, for FFPE samples (Fig. 2b).

**Fig. 3:**
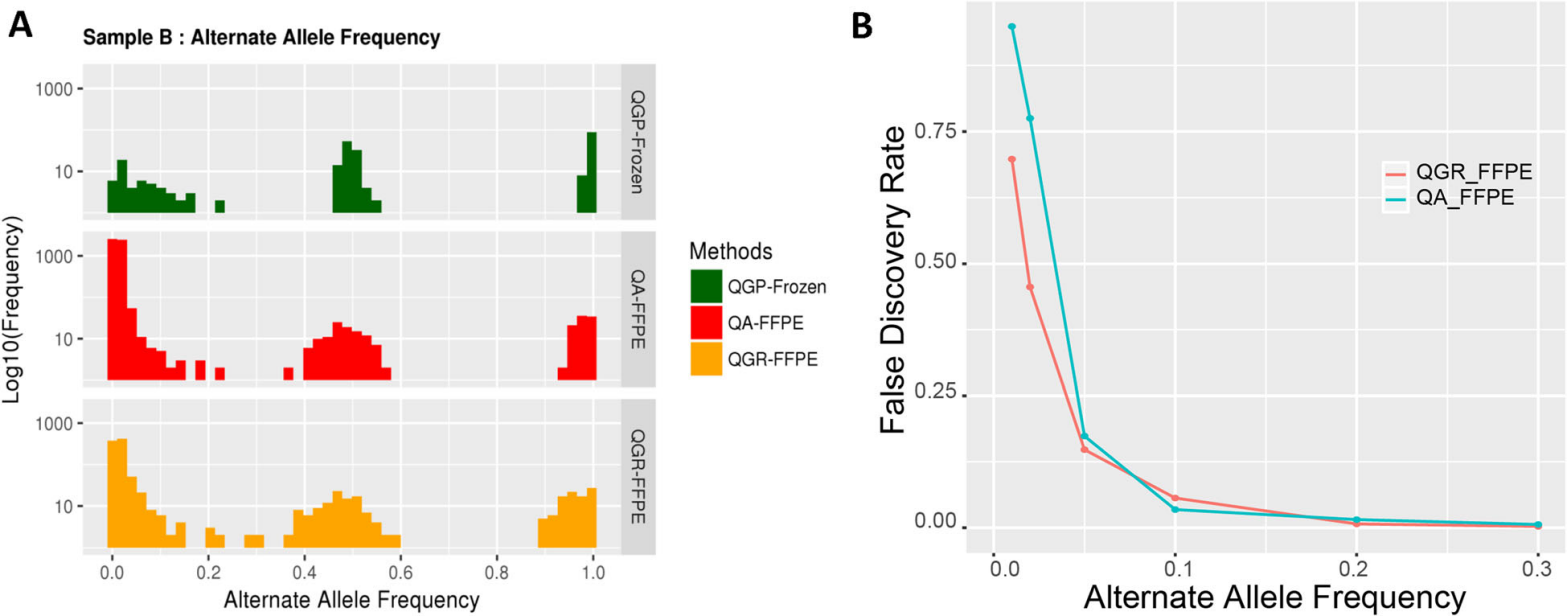
**a**. Histograms of called variants’ allele frequency in fresh-frozen and FFPE samples of subject- “B”. **b**. Estimation of false-discovery rate versus alternative allele frequency, according to variant concordance between paired fresh-frozen and FFPE samples

When we examined total number of called variants, even after the applied filtering strategy of the QIAGEN bioinformatics pipeline [8], we found that QGR-FFPE samples have approximately five times more called variants than their frozen pairs; QA-FFPE samples have even larger numbers of variants than QGR-FFPE samples (Additional file 2). Examination of allelic frequency of called variants revealed that a number of called-variants were enriched in the zone of low alternative allele frequency (AAF) (percentage of variants with AAF < 5%: 76% for QGR-FFPE, 94% for QA-FFPE), shown in Fig. 3a as an example. While this may represent true mosaic variants from a small portion of cells, we found that most variants with low AAF were discordant between FFPE and frozen pairs (Fig. 3b); in particular, the estimated FDRs for QA-FFPE and QGR-FFPE samples are 94.8 and 69.8% with variants with AAF below 1%. The fact that QGR-generated sequencing led to a smaller number of variants and lower FDR suggests the validity of DNA repair enzyme uracil N-glycosylase in the QGR method, which aids in the removal of uracils caused by cytosine deamination during formalin fixation. Left untreated, uracils are read as C > T transitions during sequencing. FDRs tend to dramatically reduce with an increase in the AAF cutoff, e.g. with AAF 5% or above, both DNA extraction methods had FDR lower than 20% (FDR = 14.8% for QGR, 17.4% for QA).

**Fig. 4:**
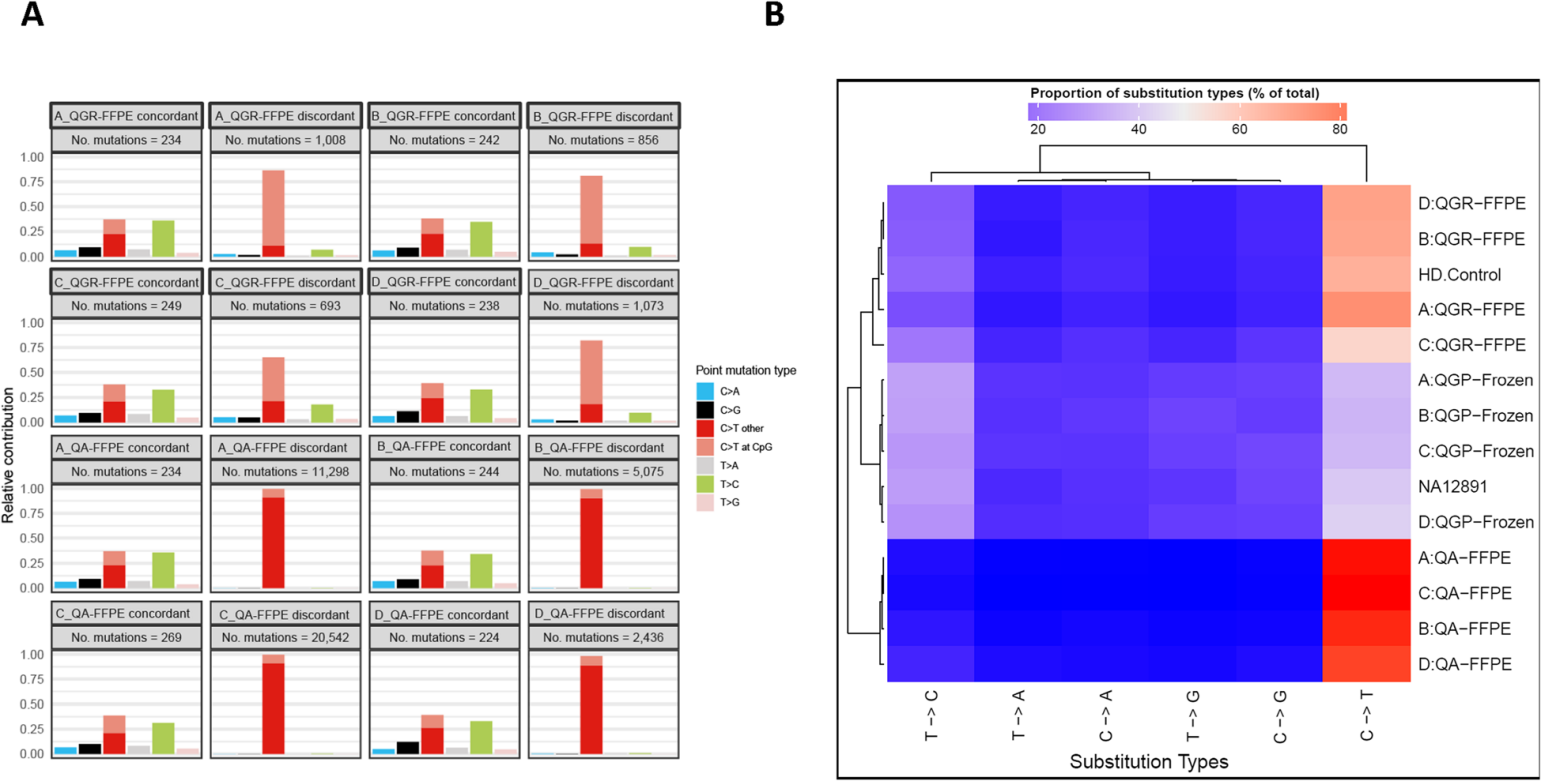
**a.** Substitution frequency bar-plot for all the samples. **b.** clustering heatmap of substitution frequencies across samples. The QA-FFPE samples show high proportion of C:G > T:A substitutions

When examining the substitution frequency of called variants across these samples (Fig. 4a and Additional file 3), the highest percentage of C > T transition rates was seen in QA-FFPE samples (93–98%), and while QGR-FFPE samples had lower C > T transition rates (58–77%), both were significantly higher than their frozen pairs (37–42%), chi-squared test *p* < 1e-16). When clustering all the samples according to substitution frequencies, shown as a heatmap in Fig. 4b, three clearly separated groups were formed based on DNA extraction methods: QGR, QGP and QA, indicating dominant effects of sample quality and library preparation steps on variant calling. In particular, FFPE samples had overall higher C > T rates than frozen samples, consistent with known artifacts for clinical archived tissues [4].

**Fig. 5:**
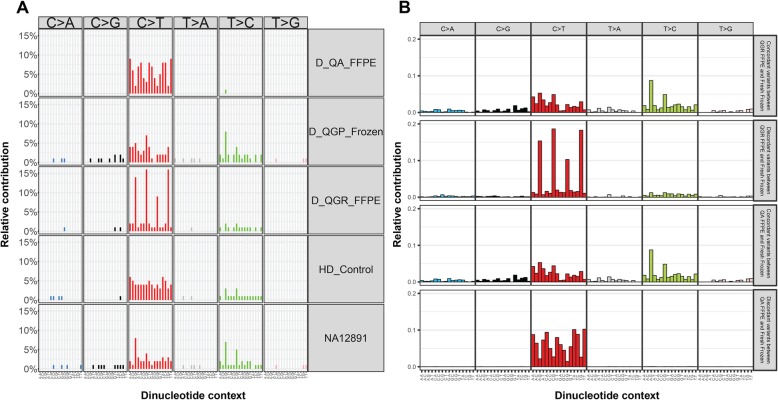
Mutational signatures according to dinucleotide context distributions of called variants. **a**. Mutational signatures according to different DNA extraction and sample types. **b**. Mutational signatures according to concordant and disconcordant variants between paired fresh-frozen and FFPE samples, respectively

In order to investigate the mutational footprints for FFPE and frozen samples, we summarized the so-called mutational signatures according to trinucleotide content around called variant positions [9]. As shown in Fig. 5a, each type of sample had unique mutational signatures: even though both QA-FFPE and QGR-FFPE samples had elevated C > T rates compared to frozen samples, their detailed spectrums are different. When variants were categorized according to FFPE versus fresh-frozen concordance status (Fig. 5b), concordant variants’ signatures had much lower proportions of C > T transitions and were more similar to the signature of the negative control (NA12891); on the other hand, the signatures of discordant variants highlighted the extraction differences between QA and QGR protocols. These signatures reflective of concordant/discordant variants were also highly similar on the individual subject level (Additional file 4), and reproducible according to de-novo mutational signature analysis (Additional file 5).

To examine sensitivity of variant calling in terms of allelic frequency, we evaluated observed versus expected frequency in a series of spike-in variants in HD sample (shown as Additional file 6**)**. All of the spike-in variants in amplicon regions were detected, with the lowest variant detectable at 1% of expected allelic frequency (EGFR T790 M mutation, expected f = 1.00%, observed f = 1.59%). Selected examples of detected variants and their allele coverage plots (demonstrated in Additional files 7, 8, and 9**)**, reflective of representative high, intermediate and low AAF variants. Together, this suggests that the mutation calling derived from the QIAGEN molecular tagging technique may be very sensitive.

## Discussion

In this study we compared variant calls from paired fresh frozen and FFPE samples. FFPE samples are generally highly degraded and thus pose significant challenges to bioinformatics analysis, especially for assessing true unbiased coverage and for calling and filtering SNPs and indels. To determine the effects of sample preparation on variant calling and downstream analysis we designed a pilot study, which included within subject replicates from breast tissue extracted using two different FFPE extraction methods.

In comparison of paired fresh frozen and FFPE breast DNA using this approach, we found that 1) it is feasible to carry out NGS studies using FFPE using a molecular barcode approach, and 2) the means by which DNA is extracted from archival tissues have an effect on NGS sequencing reads diversity. The molecular tag coverage was found to be significantly lower in the FFPE preparations, with QGR-FFPE having more duplicated reads than QA-FFPE.

3) In addition, QA-FFPE had 10-100x more variants called than did QGR-FFPE DNA. Most discordant calls between FFPE and fresh frozen derived DNA were found to be in the low allele frequency range and were more likely to occur as CG to TG transitions for both extraction methods. When comparing variant calls from paired FFPE and fresh frozen samples, the majority of the discordant and likely false-discovery variants occur in low allele frequency range (< 5%). If a simple filtering criterion is applied to exclude variants below 5% alternative allele frequency, we can achieve lower FDRs for both DNA extraction methods. 4) When examining genomics context, discordant variants were found enriched for C > T substitutions. With a dinucleotide context decomposition of called variants, we observed different mutational signatures, which have implications for deamination events and the correction step in specific DNA extraction method. However, as artefacts induced by cytosine deamination are not completely ameliorated, there are improvements to be made in this arena. 5) A spike-in positive control sample was evaluated for variant detection sensitivity, and indicative of good sensitivity of molecular barcode-based technique to confidently detect all of the spike-in variants with allele frequency as low as 1%.

Using a molecular barcode approach has been recommended as one strategy to improve sequencing results by addressing biased amplification and PCR duplication rates, which are common when low input and fragmented DNA is used that is typical from formalin fixed tissues [10]. In this approach, a unique sequence barcode is attached to each original DNA (or RNA) molecule and sequence reads having the same barcode are identified as PCR duplication events originating from the same DNA molecule. The coverage bias arising from PCR duplications of the same DNA fragment can be corrected by collapsing sequencing reads having the same molecular barcode into one single consensus read, as these reads essentially represent the same fragment. If this approach is not used, false variant calls and duplicate reads may become overrepresented in the final set of variant calls. A barcode aware analysis pipeline as provided by QIAGEN Gene Globe Portal performs optimal barcode recognition and variant calling. However, despite the consensus read approach, there are usually still a very high number of variant calls in these FFPE samples, especially in the low frequency range. Tissue used in this study passed quality and quantification standards but it is noted that FF fragment size was on average 5x larger and contained approximately 80% more DNA per sample than their FFPE counterparts. In addition, FFPE DNA has lower molecular abundance than does fresh frozen DNA, reflected in lower molecular tag coverage **(**Fig. 2**)**. However, using molecular barcoding does allow improved variant calling, and indeed this is being adopted by several molecular biology companies, such as Agilent, Swift Biosciences and Archer DX.

Others have reported use of FFPE DNA in NGS applications [3, 11–15]. Spencer et al. compared clinical NGS sequencing data from unpaired FFPE and fresh frozen DNA samples using a capture based approach [3]. Overall, they observed fewer variants in the FFPE samples than observed in this report when considering high confidence calls with high mapping quality requirement (smCounter uses a similar strategy), and the discrepant variants were but a small fraction. The FFPE samples used in that study (from lung cancer) were of reasonable quality, amplifying 200 bp fragments and yielding abundant DNA. In the present report, few if any of the breast tissue FFPE samples would have passed the amplification metric, having most of the DNA < 200 bp. Furthermore, as DNA yields were low, we used a low input sequencing approach (20–40 ng).

The use of paired fresh frozen samples and positive/negative control samples can help evaluate the accuracy and FDRs of these calls. In cases where paired fresh frozen tissue is not available, more filtering criteria (e.g. higher allele frequency), careful designs, and additional validations are highly recommended to verify artefactual variant findings from FFPE samples. Based on the findings of this study, we recommend QGR over QA as a better DNA extraction method for FFPE DNA sequencing purposes, as it significantly reduced discordant variants between FFPE and frozen pairs. Even with the adoption of the QGR protocol, significantly higher amounts of C > T transitions are still seen in FFPE samples likely due to cytosine deamination. Given the findings from the FFPE-frozen comparison that most of the discordant variants were enriched in the low allelic frequency range (less than 5%), we suggest that low frequency (1–4%) variants should be interpreted with caution and subjected to further filtering, particularly when using very fragmented DNA. Because the detection sensitivity of the QIAGEN barcoding panel can be as low as 1%, according to our examination of spike-in positive control samples, we recommend a hybrid strategy of filtering out low allelic frequency variants and prioritizing likely pathogenic variants according to bioinformatics annotations.

Based on this study, we believe at least three design strategies can be generalized to other research of calling variants from FFPE DNA-seq data: (1) A study design including replicates (ideally of FFPE versus fresh samples, or at least independent FFPE replicates) is essential to understand intrinsic concordance and reproducibility of variant calling. (2) FFPE DNA-seq samples are prone to low allele frequency artifacts, which sometimes may be mistakenly taken as mosaic variants. Therefore, false discovery assessments according to variant allele frequency are highly recommended. (3) Certain mutational signatures (e.g. C > T) are significantly associated with FFPE samples independent of DNA extraction protocols. Decomposition of mutational signatures is a valuable QC procedure to highlight problematic FFPE samples, and provides additional sample-level covariates (i.e. mutational signature activities) for further comparison and association studies across samples. Other than these above-mentioned considerations, additional FFPE quality factors (e.g. Archived age of FFPE block, initial DNA yield) and bioinformatics metrics may need to be considered and evaluated specific to each individual study.

## Conclusions

Despite significant challenges of FFPE-derived NGS data, we demonstrated that a combination of strategies, including use of molecular barcodes, optimization of the DNA extraction process, and bioinformatics variant detection and filtering strategies, allow sensitive and confident variant calling for FFPE samples. Our results support the feasibility of using FFPE samples for variant calling and potential variant-disease association studies. In future work, we would like to generalize our filtering strategies and further investigate the nature of putative artefactual sequencing variants in larger FFPE sequencing cohorts.

## Methods

### Tissue samples

Institutional Review Board approval was obtained for research use of human samples in this project (#IRB 75–87). We selected FFPE breast tissue samples from four women who had undergone breast surgery with benign findings, with concomitant fresh frozen benign breast tissue collected on the same date from the same breast, which was stored in the institutional frozen tissue bank. Ages of these women were 41, 43, 44, and 55. Three women underwent bilateral mastectomy for risk reduction due to a strong family history of breast cancer, all with an affected first degree relative. Among these three, two underwent genetic testing for BRCA1 and BRCA2 genes with negative results. The fourth woman underwent bilateral reduction mammoplasty for symptom relief. All tissue samples used for sequencing (both fresh frozen and FFPE) were selected from the left breast. The pathology reports on these breast tissues identified cysts, apocrine metaplasia, and proliferative changes to include usual ductal hyperplasia and sclerosing adenosis. In addition to the four human subjects, positive and negative controls were included. A negative control sample with publicly available data on whole-genome sequencing (NA12891, from Coriell Institute for Medical Research, Camden, NJ, USA) was included for evaluating genotype accuracy. A positive reference control sample was used that includes 11 mutations with varying allelic frequencies from formalin treated cell line DNA to mimic degrees of degradation (Quantitative Multiplex Formalin Compromised [Moderate] Reference Standard, Horizon Discovery Cambridge, UK). This positive control that mimics FFPE DNA quality was chosen to evaluate sensitivity of detecting variants at different allelic frequencies.

### DNA extraction

For each of four subjects (labeled A-D), triplicate tissue samples were obtained: one fresh frozen tissue sample from the institutional cryobank, and two paired samples (from the same individuals) were derived from a single FFPE block (to evaluate two different DNA extraction methods from FFPE). Ten micrometer sections were cut from each block of FFPE or fresh frozen tissue using a standard microtome (Leica Rotary Microtome RM2235, Leica Biosystems, Buffalo Grove, IL). Both FFPE and fresh frozen sections were placed in microcentrifuge tubes with FFPE sections remaining at room temperature and fresh frozen tubes kept at − 20 °C until the time of extraction. All benign breast disease FFPE sections underwent DNA extraction with two methods according to manufacturer’s guidelines: QIAGEN’s GeneRead DNA FFPE kit (QIAGEN), and QIAGEN’s QIAamp DNA FFPE Tissue kit (with one modification, where the 1 h lysis incubation at 56 °C was replaced with an overnight incubation at the same temperature). The paired benign breast disease fresh frozen samples underwent DNA extraction using QIAGEN’s Gentra Puregene Kit following manufacturer’s guidelines for DNA Purification from Tissue. Both positive and negative controls were extracted by their respective companies using the Promega Maxwell LEV Plus FFPE (HD799) and QIAGEN Autopure LS (NA12891). After extraction, DNA was quantified using Qubit™ dsDNA BR Assay (ThermoFisher Scientific, Waltham, MA, USA) while quality was assessed using the Advanced Analytical Fragment Analyzer™ High Sensitivity Large Fragment Analysis kit which calculates fragment length and degradation.

### Library preparation and sequencing

The QIAseq Human Breast Cancer Targeted Panel was chosen for this trial as it targets 93 genes relevant in breast cancer. To prepare libraries we followed QIAGEN’s guidelines for FFPE DNA. As per manufacturer input guidelines, 20–40 ng of DNA from the extracted FFPE, fresh frozen and control samples were enzymatically fragmented, end repaired and A-tailed in a 25 μl reaction mix which contained 2.5 μl of 10x fragmentation buffer, 0.75 μl of FERA solution and 5 μl of Fragmentation Enzyme Mix. The reaction mix was incubated at 4 °C for 1 min, 32 °C for 14 min, and 72 °C for 30 min. After incubation, the reaction was placed on ice and 10 μl of 5x Ligation Buffer, 2.8 μl of barcode adapter, 5 μl of DNA Ligase and 7.2 μl of Ligation solution were added and mixed by pipet. The reaction was placed at 20 °C for 15 min. Reactions were purified using two 1.0 X QIAseq Bead clean up steps. After purification, 4 μl of 5X TEPCR buffer, 5 μl of QIAseq Human Breast Cancer Panel, 0.8 μl of IL-Forward primer and 0.8 μl of HotStar Taq DNA Polymerase were added to the 9.4 μl of adapter ligated DNA. Enrichment PCR conditions were: 95 °C for 13 min, 98 °C for 2 min; 6 cycles of 98 °C for 15 s and 65 °C for 30 min; 72 °C for 5 min. Target enrichment was purified using a 1.0X QIAseq Bead clean up. Purified target enriched DNA was mixed with 4 μl of 5X UPC Buffer, 0.8 μl IL-Universal Primer, 0.8 μl IL-Index primer, and 1 μl HotStar Taq DNA polymerase for a total of 20 μl. Universal PCR conditions were: 95 °C for 13 min, 98 °C for 2 min; 24 cycles of 98 °C for 15 s and 60 °C for 2 min; 72 °C for 5 min. After the reaction was complete the Universal PCR reaction was purified using a 1.0X QIAseq Bead clean-up. Libraries were quantified and sequenced on the Illumina HiSeq 4000, paired end 150-bp.

### Analysis

The QIAGEN Gene Globe Data Portal [16] was used for analysis of the samples. For analysis, the raw FASTQ files were uploaded to the data portal. The QIAGEN analysis steps includes adapter trimming, coupling molecular tag (MT) sequence to the read IDs, alignment to the reference genome and subsequent variant calling using smCounter, QIAGEN’s molecular tag-aware variant calling algorithm. smCounter uses a Bayesian probabilistic model to identify variants and infer genotypes, and can detect low frequency variants with high sensitivity. Since each unique DNA fragment generated from the experiment should have a unique molecular tag attached, sequenced reads with identical molecular tags were identified as PCR duplicates. During analysis, such reads arising from PCR duplicates were collapsed to create a consensus read sequence. In order to reflect molecular diversities of each sample, a so-called molecular diversity score was defined as proportion of molecular-tag coverage versus raw sequencing coverage (100 x MT-coverage/Raw-coverage). For variant calling, the consensus sequence was compared to the reference genome. The GRCh37 build of the human genome was used as the reference for this analysis. For variant calling, the smCounter algorithm calculates a prediction index of the alleles observed at the barcode level for every target position and a variant is called if an allele shows a higher prediction index compared to a preselected prediction index threshold. This threshold is based on the demonstration by smCounter that 8 reads per barcode is sufficient for variant analysis as described in [8]. The output from the variant calling step was obtained in the standard variant calling format (VCF). After variant-calling, initial variant filtering was done to exclude likely false calls due to technical factors, such as presence in low complexity regions, shallow molecular tag coverage, strand bias, and/or low base quality. Only variants passing filter recommendations by the QIAGEN bioinformatics pipeline were used for subsequent analysis.

The variants passing all aforementioned filters were then used for calculating sample-level genotype similarity score, which was defined as correlation of variant alternate allele frequencies of paired samples from the known single-nucleotide polymorphisms (SNPs) within the panel [17]. Sample level genotype similarity scores were computed across all possible paired samples to ensure subject identity, using variants having at least 50X molecular tag coverage. Additionally, variant-level concordance was evaluated if the genotypes defined by smCounter were identical for the FFPE-Fresh Frozen pair used for comparison: identical genotypes mean concordant, otherwise discordant for this variant position. The genotype concordance rate of the control NA12891 sample was assessed by comparing the variant calls to the “ground truth” calls derived from publicly available NA12891 whole genome sequencing (WGS) sample. For the Horizon Discovery (HD) control sample, the pre-determined expected allele frequencies were compared to the observed allele frequencies in the sample obtained from the sequencing data for approximate equivalence.

Additionally, a set of custom Perl scripts were used to generate a mutation frequency table of all single nucleotide variants SNVs across each sample to assess the type of mutations being reported. This mutation frequency table was subsequently used to generate plots of mutation signatures for the variants using Perl and R scripts. Sample identity was examined using NGSCheckmate (version 1.3) [17], which uses depth-dependent correlation models of allele fractions of known SNPs to identify samples from the same individual. Mutational spectrums and de-novo mutational signatures were identified using MutationalPatterns package (version 1.2.1). De-novo signatures were extracted based on non-negative matrix factorization (NMF) algorithm.

## Additional files

Additional file 1: Sample identity checks according to genotype concordance using NGScheckmate (TIFF 849 kb)

Additional file 2: Table showing NGS metrics for all the study samples (DOCX 14 kb)

Additional file 3: Table showing substitution frequency distributions for all the study samples (DOCX 15 kb)

Additional file 4: Per sample mutational signature for called variants (TIFF 539 kb)

Additional file 5: De-novo mutational signatures (TIFF 1314 kb)

Additional file 6: Table showing expected versus observed variant allelic frequency in positive control samples (HD) (DOCX 18 kb)

Additional file 7: Selected example of relatively high allelic frequency of mutation detected in positive control sample (PIK3CA H1047R). (TIFF 361 kb)

Additional file 8: Selected example of intermediate allelic frequency of mutation detected in positive control sample (MLH1 L323) (TIFF 336 kb)

Additional file 9: Selected example of low allelic frequency of mutation detected in positive control sample (EGFR T790 M) (TIFF 354 kb)

## Data Availability

Raw sequencing data are available from the corresponding author on reasonable request.

## Abbreviations

AAF: Alternative allele frequency
FDR: False discovery rate
FFPE: Formalin fixed paraffin embedded
HD: Horizon discovery
MT: Molecular tag
NGS: Next generation sequencing
NMF: Negative matrix factorization
QA: QIAGEN QIAamp DNA Mini Kit
QGP: Qiagen Gentra Puregene Kit
QGR: QIAGEN GeneRead DNA FFPE Kit
SNV: Single-nucleotide variants
VCF: Variant calling format
WGS: Whole genome sequencing
