# Supplementary figures and images for: Bioinformatics and DNA-extraction strategies to reliably detect genetic variants from FFPE breast tissue samples
Source: BMC Genomics. 2019 Sep 2;20:689. doi: 10.1186/s12864-019-6056-8 (PMC6720378; doi:10.1186/s12864-019-6056-8)

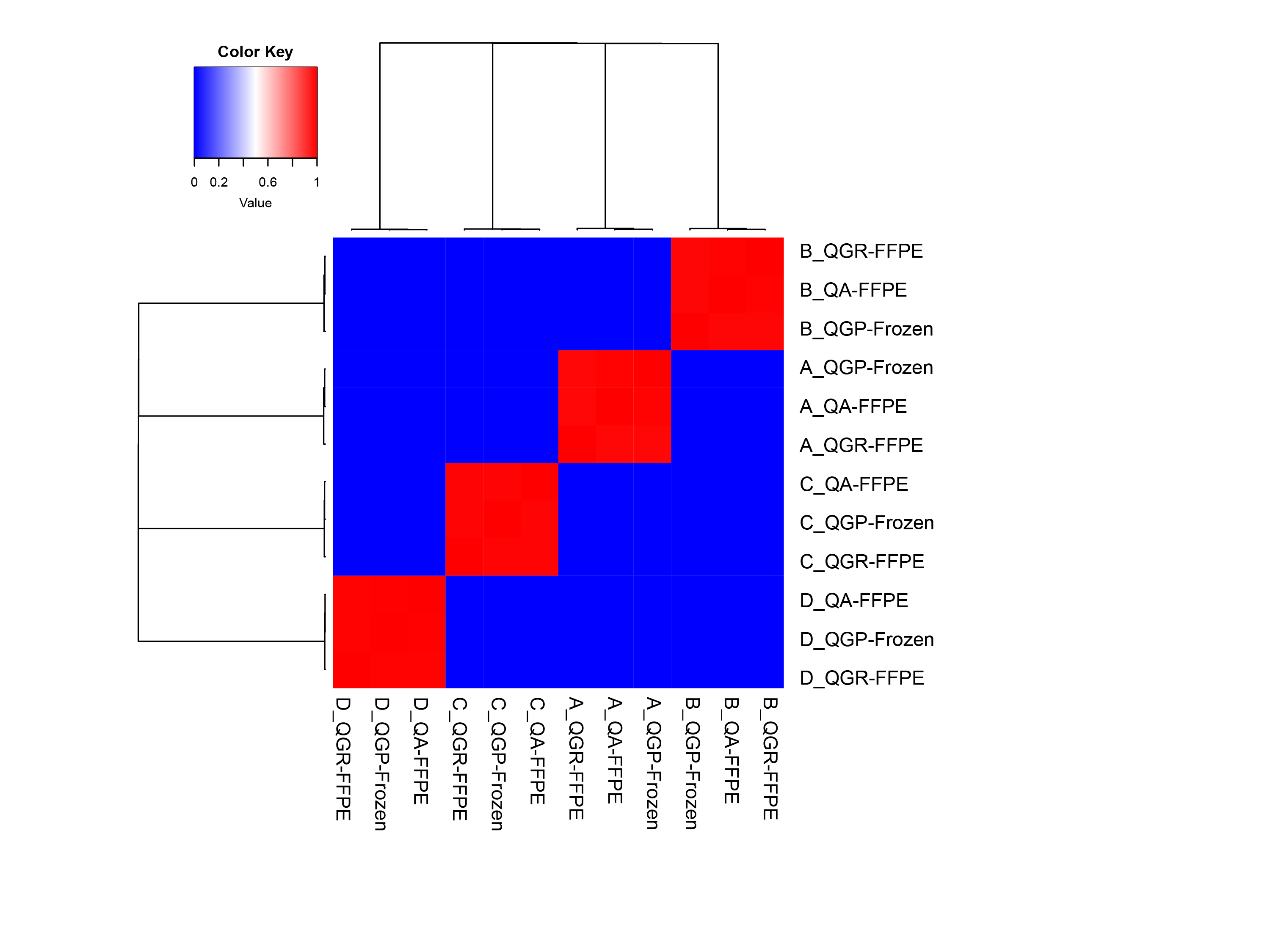

Supplement: Supplementary file 1 — Sample identity checks according to genotype concordance using NGScheckmate (TIFF 849 kb) [file 12864_2019_6056_MOESM1_ESM.tiff]

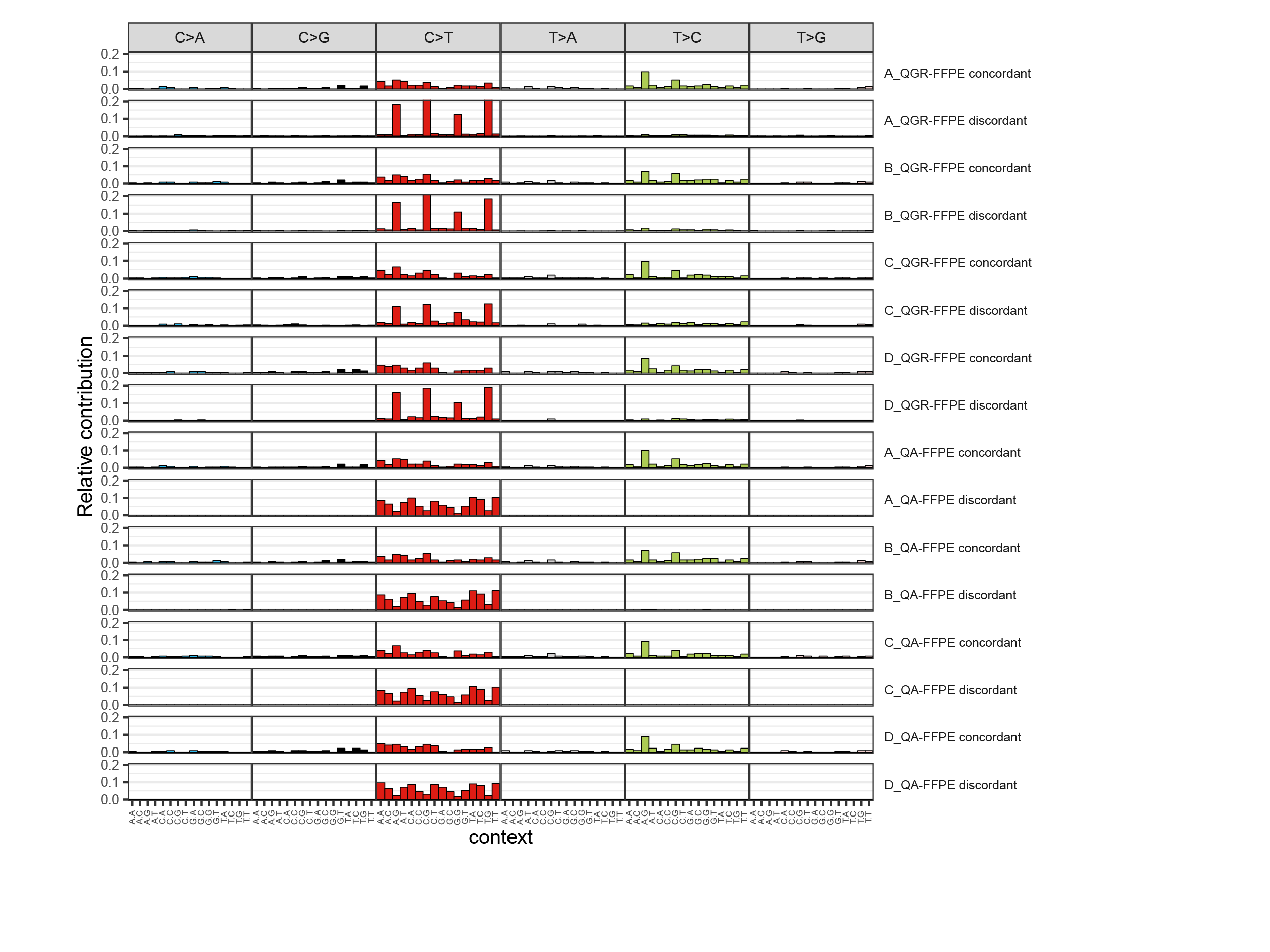

Supplement: Supplementary file 4 — Per sample mutational signature for called variants (TIFF 539 kb) [file 12864_2019_6056_MOESM4_ESM.tiff]

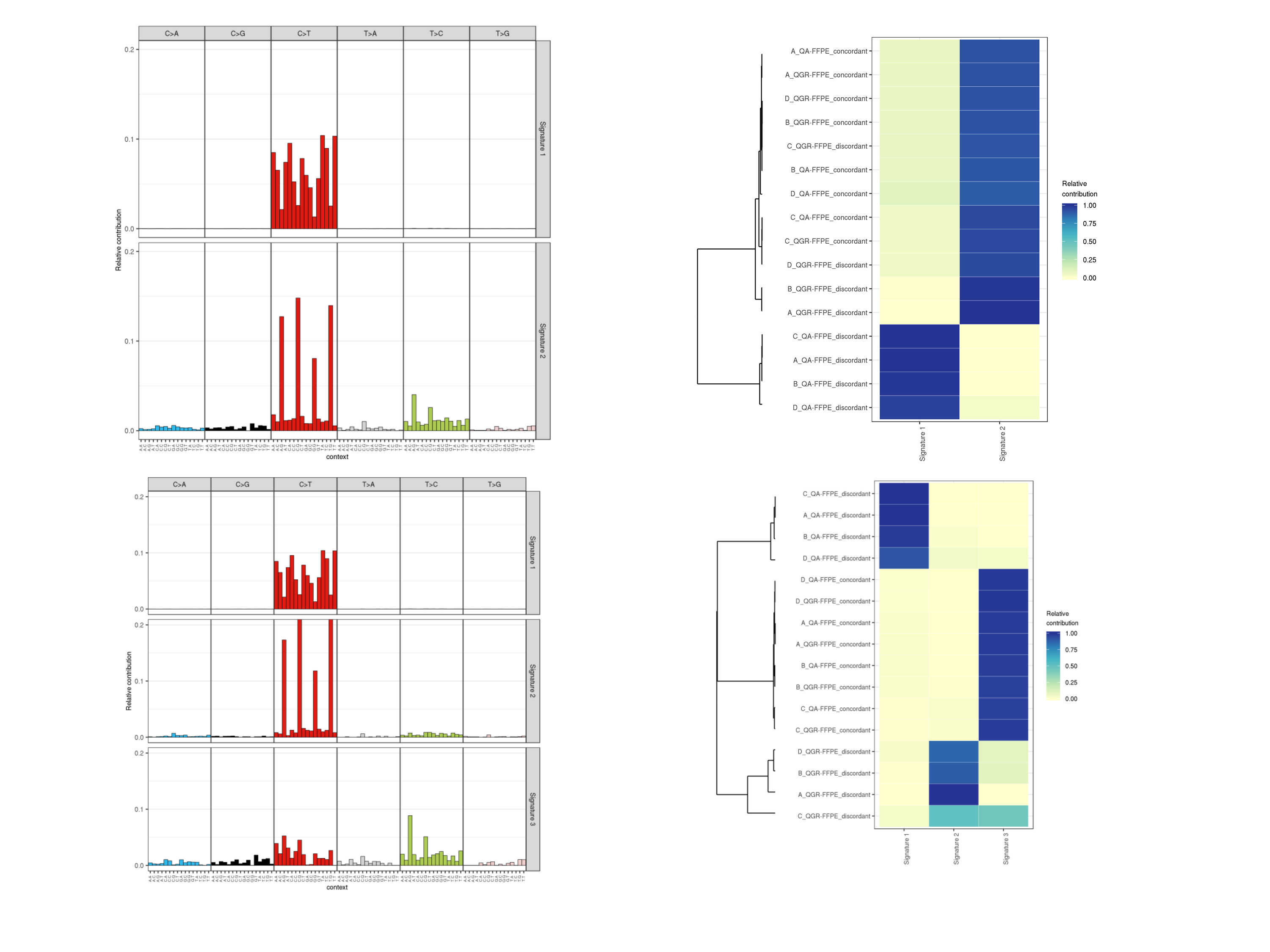

Supplement: Supplementary file 5 — De-novo mutational signatures (TIFF 1314 kb) [file 12864_2019_6056_MOESM5_ESM.tiff]

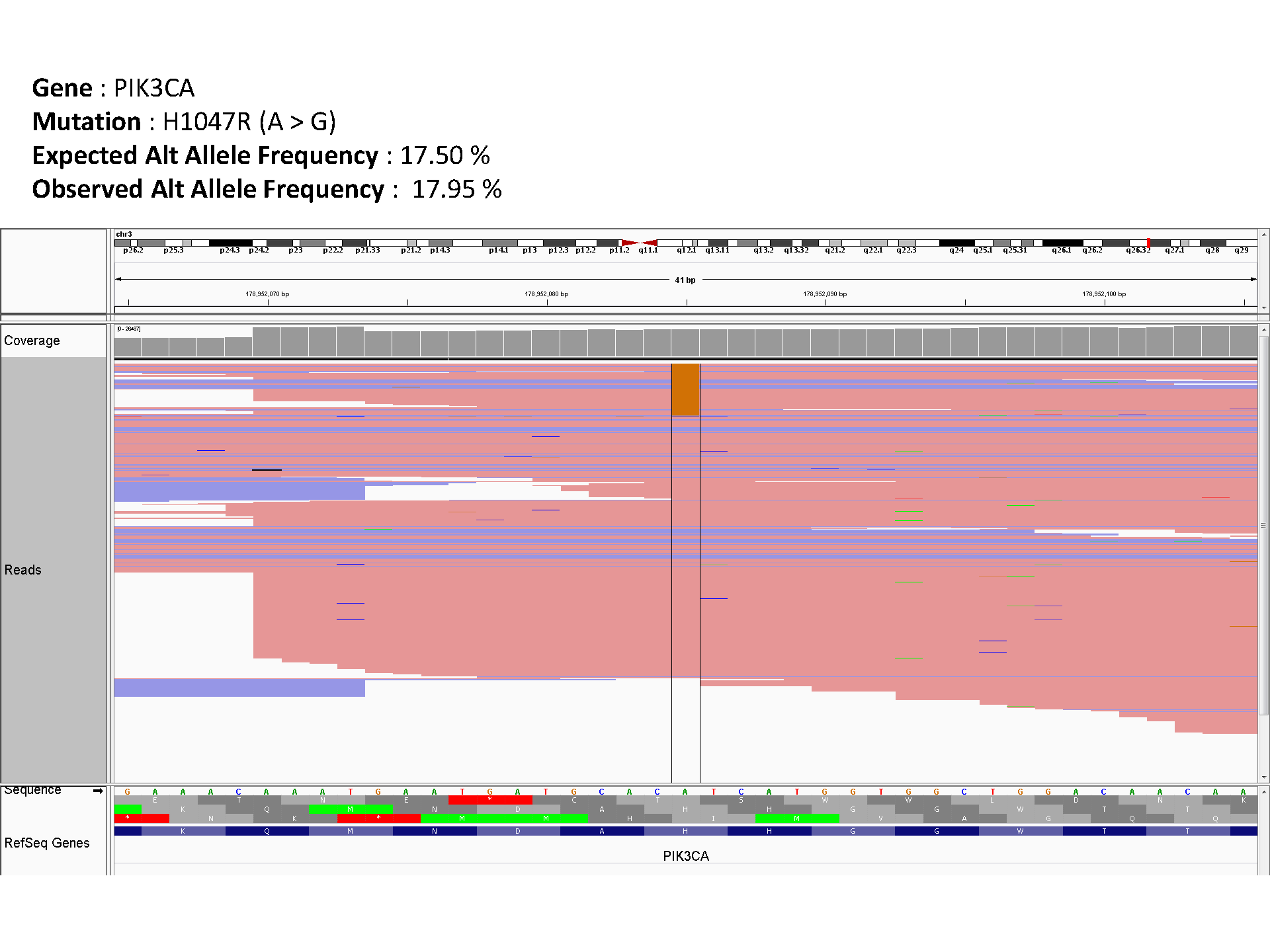

Supplement: Supplementary file 7 — Selected example of relatively high allelic frequency of mutation detected in positive control sample (PIK3CA H1047R). (TIFF 361 kb) [file 12864_2019_6056_MOESM7_ESM.tiff]

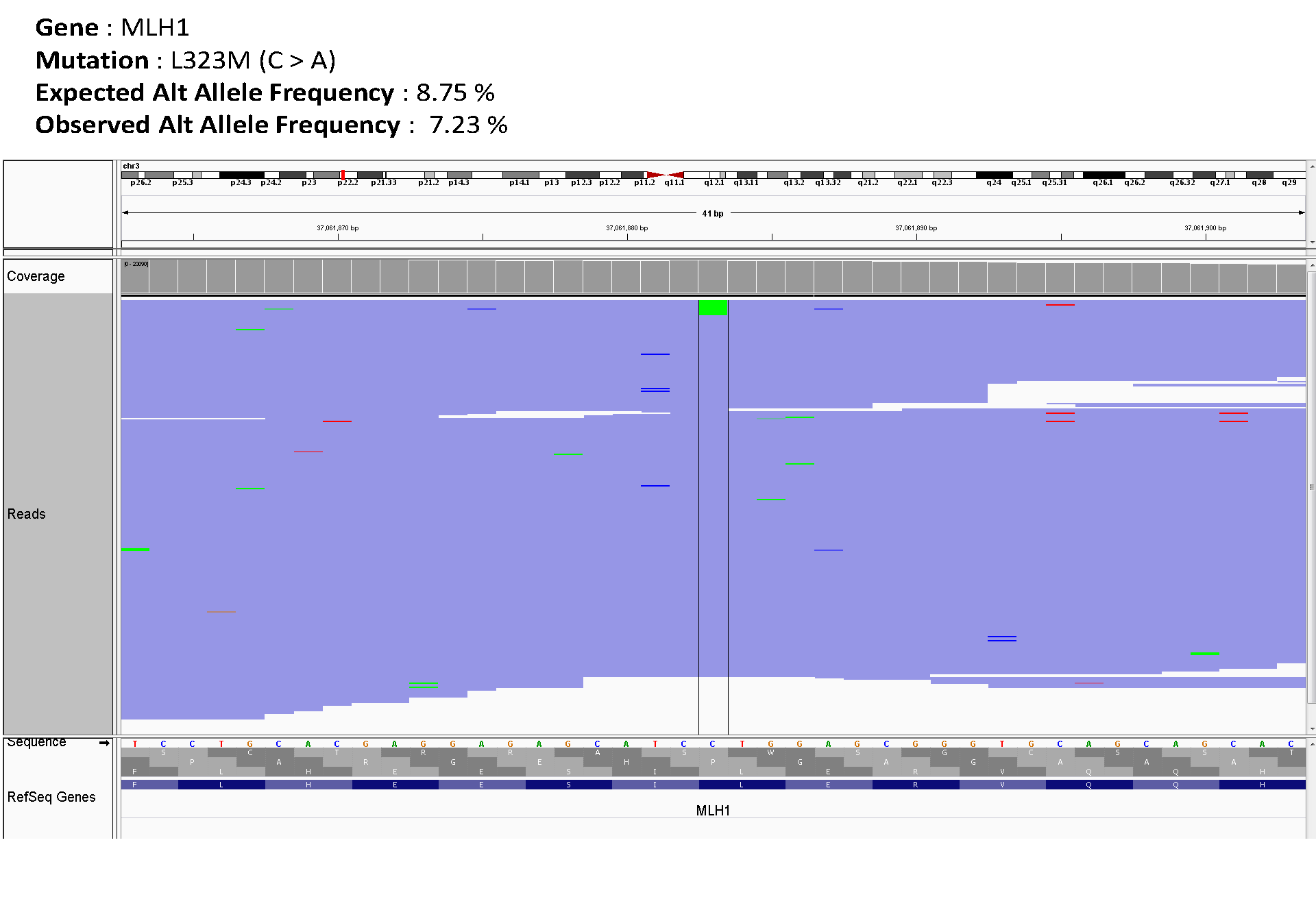

Supplement: Supplementary file 8 — Selected example of intermediate allelic frequency of mutation detected in positive control sample (MLH1 L323) (TIFF 336 kb) [file 12864_2019_6056_MOESM8_ESM.tiff]

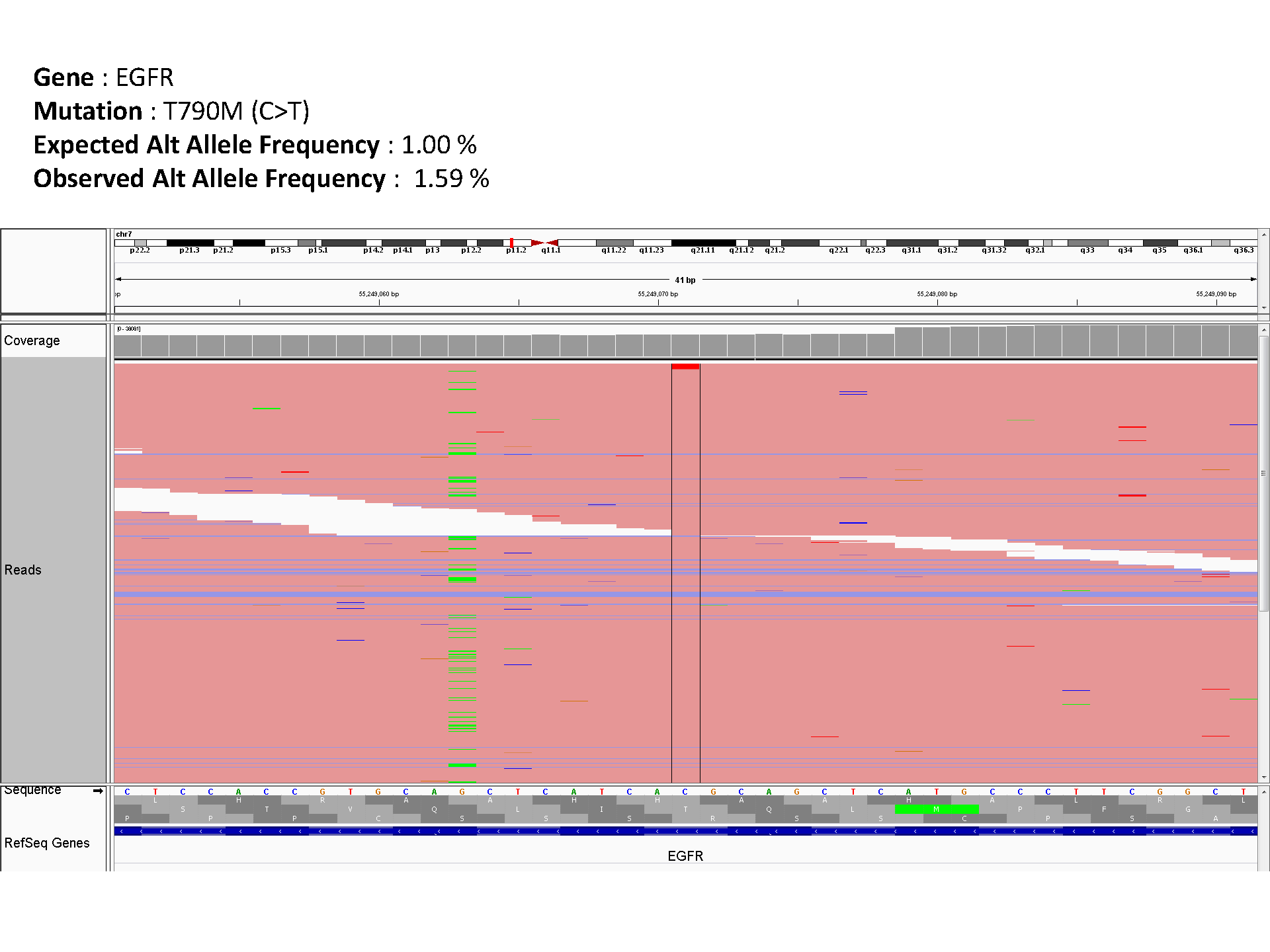

Supplement: Supplementary file 9 — Selected example of low allelic frequency of mutation detected in positive control sample (EGFR T790 M) (TIFF 354 kb) [file 12864_2019_6056_MOESM9_ESM.tiff]
