# Supplementary material for: Bioinformatics and DNA-extraction strategies to reliably detect genetic variants from FFPE breast tissue samples
Source: BMC Genomics. 2019 Sep 2;20:689. doi: 10.1186/s12864-019-6056-8 (PMC6720378; doi:10.1186/s12864-019-6056-8)
Supplement: Supplementary file 2 — Table showing NGS metrics for all the study samples (DOCX 14 kb) [file 12864_2019_6056_MOESM2_ESM.docx]

Additional File 2. NGS metrics for all the study samples

| Sample / NGS metrics | MT-covg:  Average MT read-depth | Raw covg:  Average Raw NGS Read-Depth | Molecular Diversity Score = 100x (MT-covg/Raw-Covg) | N. of called variants |
| --- | --- | --- | --- | --- |
| A_QGP-Frozen | 2188.6 | 12902.6 | 16.96 | 248 |
| A_QA-FFPE | 423.3 | 12559.4 | 3.37 | 11533 |
| A_QGR-FFPE | 400.5 | 15243.2 | 2.63 | 1247 |
| B_QGP-Frozen | 1479 | 10225.9 | 14.46 | 262 |
| B_QA-FFPE | 414.1 | 8424.8 | 4.91 | 5318 |
| B_QGR-FFPE | 511.7 | 10058.3 | 5.09 | 1108 |
| C_QGP-Frozen | 1645.3 | 22209.8 | 7.41 | 291 |
| C_QA-FFPE | 578.6 | 19327 | 3 | 20820 |
| C_QGR-FFPE | 223.6 | 6062.9 | 3.69 | 927 |
| D_QGP-Frozen | 1294.9 | 17424 | 7.43 | 267 |
| D_QA-FFPE | 179.2 | 17562.1 | 1.02 | 2639 |
| D_QGR-FFPE | 478.2 | 18152.6 | 2.63 | 1316 |
