# Supplementary material for: Bioinformatics and DNA-extraction strategies to reliably detect genetic variants from FFPE breast tissue samples
Source: BMC Genomics. 2019 Sep 2;20:689. doi: 10.1186/s12864-019-6056-8 (PMC6720378; doi:10.1186/s12864-019-6056-8)
Supplement: Supplementary file 3 — Table showing substitution frequency distributions for all the study samples (DOCX 15 kb) [file 12864_2019_6056_MOESM3_ESM.docx]

**Additional Table 2**. Substitution frequency distributions for all the study samples

| Sample / Substitution | C>A | C>G | C>T | T>A | T>G | T>C | Total N. of Variants |
| --- | --- | --- | --- | --- | --- | --- | --- |
| **A_QGP-Frozen** | 16  (6.64 %) | 22 (9.13 %) | 90  (37.34 %) | 17 (7.05 %) | 9 (3.73 %) | 87 (36.10 %) | 241 |
| **A_QA-FFPE** | 18  (0.16 %) | 29 (0.25 %) | 11338 (98.38 %) | 20 (0.17 %) | 13 (0.11 %) | 107 (0.93 %) | 11525 |
| **A_QGR-FFPE** | 41  (3.32 %) | 40 (3.24 %) | 955  (77.33 %) | 25 (2.02 %) | 23 (1.86 %) | 151 (12.23 %) | 1235 |
| **B_QGP-Frozen** | 18  (7.09 %) | 22 (8.66 %) | 97  (38.19 %) | 19 (7.48 %) | 12 (4.72 %) | 86  (33.86 %) | 254 |
| **B_QA-FFPE** | 26  (0.49 %) | 22 (0.41 %) | 5130 (96.63 %) | 18 (0.34 %) | 14 (0.26 %) | 99  (1.86 %) | 5309 |
| **B_QGR-FFPE** | 49  (4.51 %) | 42 (3.87 %) | 780  (71.82 %) | 24 (2.21 %) | 25 (2.30 %) | 166  (15.29 %) | 1086 |
| **C_QGP-Frozen** | 20  (7.14 %) | 27 (9.64 %) | 108  (38.57 %) | 24 (8.57 %) | 14 (5.00 %) | 87  (31.07 %) | 280 |
| **C_QA-FFPE** | 28  (0.13 %) | 40 (0.19 %) | 20533 (98.72 %) | 29 (0.14 %) | 23 (0.11 %) | 147  (0.71 %) | 20800 |
| **C_QGR-FFPE** | 50  (5.49 %) | 56 (6.15 %) | 531  (58.29 %) | 40 (4.39 %) | 34 (3.73 %) | 200  (21.95 %) | 911 |
| **D_QGP-Frozen** | 15  (5.86 %) | 27 (10.55 %) | 110  (42.97 %) | 16 (6.25 %) | 10 (3.91 %) | 78  (30.47 %) | 256 |
| **D_QA-FFPE** | 16  (0.61 %) | 30 (1.14 %) | 2456 (93.46 %) | 24 (0.91 %) | 11 (0.42 %) | 91  (3.46 %) | 2628 |
| **D_QGR-FFPE** | 47  (3.63 %) | 47 (3.63 %) | 957  (74.01 %) | 33 (2.55 %) | 27 (2.09 %) | 182  (14.08 %) | 1293 |
