# Supplementary material for: Bioinformatics and DNA-extraction strategies to reliably detect genetic variants from FFPE breast tissue samples
Source: BMC Genomics. 2019 Sep 2;20:689. doi: 10.1186/s12864-019-6056-8 (PMC6720378; doi:10.1186/s12864-019-6056-8)
Supplement: Supplementary file 6 — Table showing expected versus observed variant allelic frequency in positive control samples (HD) (DOCX 18 kb) [file 12864_2019_6056_MOESM6_ESM.docx]

**Additional Table 3.**  Expected versus observed variant allelic frequency in positive control samples (HD)

| **Chromosome** | **Gene** | **Variant** | **Expected Allelic Frequency, %** | **Observed Allelic Frequency, %** | **Chromosome Location** |
| --- | --- | --- | --- | --- | --- |
| 7q34 | BRAF | V600E | 10.50% | Not Targeted By Panel | chr7 140453136 |
| 4q11-q12 | cKIT | D816V | 10.00% | Not Targeted By Panel | chr4 55599321 |
| 7p12 | EGFR | ΔE746 - A750 | 2.00% | 0.67% | chr7 55242464 |
| 7p12 | EGFR | L858R | 3.00% | 3.59% | chr7 55259515 |
| 7p12 | EGFR | T790M | 1.00% | 1.59% | chr7 55249071 |
| 7p12 | EGFR | G719S | 24.50% | 26.01% | chr7 55241707 |
| 12p12.1 | KRAS | G13D | 15.00% | 15.77% | chr12 25398281 |
| 12p12.1 | KRAS | G12D | 6.00% | 7.50% | chr12 25398284 |
| 1p13.2 | NRAS | Q61K | 12.50% | Not Targeted By Panel | chr1 115256530 |
| 3q26.3 | PIK3CA | H1047R | 17.50% | 17.95% | chr3 178952085 |
| 3q26.3 | PIK3CA | E545K | 9.00% | 4.27% | chr3 178936091 |
| 2p23 | ALK | P1543S | 33% | Not Targeted By Panel | chr2 29416326 |
| 13q12 | FLT3 | V197A | 11.50% | Not Targeted By Panel | chr13 28626706 |
| 2q33.3 | IDH1 | S261L | 10% | Not Targeted By Panel | chr2 209106786 |
| 3p21.3 | MLH1 | L323M | 8.50% | 7.23% | chr3 37061883 |
| 9q34.3 | NOTCH1 | P668S | 31.50% | Not Targeted By Panel | chr9 139409754 |
| 4q12 | PDGFRA | G426D | 33.50% | Not Targeted By Panel | chr4 55138600 |
